# Factors associated with spontaneous abortion: a cross-sectional study of Chinese populations

**DOI:** 10.1186/s12978-017-0297-2

**Published:** 2017-03-04

**Authors:** Danni Zheng, Chunyan Li, Taiwen Wu, Kun Tang

**Affiliations:** 1 Department of Global Health, School of Public Health, Peking University, Beijing, 100191 China; 2 School of Basic Medical Science, Peking University, Beijing, 100191 China

**Keywords:** Spontaneous abortion, Socioeconomic status, Maternal health, China Kadoorie Biobank

## Abstract

**Background:**

Spontaneous abortion (SA) is one of the prevalent negative reproductive outcomes among women around the world, which is a great challenge faced by maternal health promotion. The present study is aimed to explore the association between SA and socioeconomic status (SES) and provides reference for policy makers to improve strategies on maternal health promotion.

**Methods:**

A cross-sectional analysis was conducted with baseline data from a large-scale population-based cohort study of 0.5 million people from 10 geographically diverse areas of China recruited from 2004 to 2008. The study collected data from 84,531 women aged 35–45 years old in the baseline survey of China Kadoorie Biobank. Participants were interviewed using a standardized questionnaire, and information on demographic-socioeconomic as well as reproductive health status was collected. Odds ratios (OR) with 95% CI, estimated by a multistep logistic regression, were used to approximate the associations between SA occurrence and characteristics of SES. A stratification analysis was also applied to find out how SES influenced women’s reproductive health outcomes differently between rural and urban areas. The model was adjusted for age at study date, tea consumption, alcohol consumption, cigarette smoking, and number of induced abortion.

**Results:**

The risk of SA in rural was 1.68 times greater than in urban (AOR = 1.68, 95%CI: 1.54–1.84). Women with high income had a decreased risk of SA when compared with that of women with low income (AOR = 0.90, 95%CI: 0.84–0.97). Compared with women in low educational attainment, women in higher educational attainment had a lower prevalence of SA (AOR = 0.90, 95%CI: 0.82–0.98). The risk of SA only reduced in factory worker (AOR = 0.59, 95%CI: 0.53–0.66) and professional worker (AOR = 0.75, 95%CI: 0.66–0.84) compared with agriculture and related workers. After stratifying by rural/urban, the association between income and SA in urban (AOR = 0.88, 95%CI: 0.78–0.99) was stronger than that in rural (AOR = 0.92, 95%CI: 0.84–1.00). Association between education and SA was found in urban (AOR = 0.66, 95%CI: 0.55–0.78) but not in rural (AOR = 1.05, 95%CI: 0.34–1.17), and there was no difference on how occupation impacted SA among women between the two subgroups.

**Conclusions:**

Generally women with lower SES status had a higher risk of SA. Lower income and educational attainment were inversely associated with the risk of SA. Women with agricultural and related work had a significantly higher prevalence of SA. Interventions could be targeted more on women with low SES to increase both health profits as well as economic gains for health programs.

**Electronic supplementary material:**

The online version of this article (doi:10.1186/s12978-017-0297-2) contains supplementary material, which is available to authorized users.

## Plain English summary

The present study utilized the baseline data from the China Kadoorie Biobank to explore the association between socio-economic status (SES) and spontaneous abortion (SA), providing a reference for policy makers to improve maternal health strategies. Participants were interviewed using a standardized questionnaire, and information about demographic-socioeconomic as well as reproductive health status was collected. In total, 84,531 women from 10 areas in China were recruited into the study using descriptive analysis and multistep logistic regression. It was found in this study that the risk of spontaneous abortion in rural was higher than urban. Women with high income had a lower risk of SA when compared with women with low income. Compared with women with lower educational level, the risk of SA in women with higher educational level was lower. The risk of SA only reduced in factory worker and professional worker compared with agriculture and related workers. After stratifying by rural/urban, the association between income and SA in urban was stronger than rural. Association between education and SA was found in urban but not in rural, and there was no difference in occupation between two subgroups. These results provide important implications for policy and program development.

## Background

Spontaneous abortion (SA), also known as miscarriage, is considered as one of the most frequent problems a woman may encounter during early pregnancy which usually predominates from chromosomal abnormalities and hormonal problems [1–3]. It was estimated that 6–15% of all clinically detected pregnancies end with SA in overseas and the incidences of SA in China were 6–14% [4–8]. The number is thought to be much higher if the occurrence of very early pregnancy losses were considered. Some researchers in Egypt and Denmark had found higher incidence of SA over 20% in recent years [7, 9].

Socioeconomic status (SES), as indicated by level of wealth, education, and occupation, has been well established as associated with an individual’s health. Such socioeconomic factors played an influential part in the health and life expectancies of the pregnancy and impact on pregnant outcomes, for people in low SES are more likely to show risky behavioral patterns and enjoy limited access to health services [10, 11]. A great number of, even as many as abundant, studies have well established the association among SES, onset of non-communicable diseases and cancer [12, 13]. However, up till now, few studies have investigated specifically the relationship between SES and the occurrence of SA, and no consensus has been reached yet.

Low income levels were associated with poor housing, nutrition and health care access, as well as increasing the risk of spontaneous abortion. It has been investigated that women with low SES have an increased risk of SA when measured by income or wealth, while other studies found no such significant correlations [6, 14–16]. The level of educational attainment is an international indicator of social position. According to literature, maternal educational level influenced pregnancy outcomes though access to health services and care strategies for both mother and fetus [16, 17]. Some researchers have reported that there was inversely association between educational level and SA, while some studies did not find the association [14, 17–19]. Compared to income and education, employment status was controversial and information as to whether adverse effects of occupation affect spontaneous abortion was scant. A couple of studies demonstrated that employment increased the risk of SA, but more studies reported no association between employment and spontaneous abortion [10, 15, 18, 20]. In addition, lifestyle factors were significant associated with female’s reproductive health, such as tea drinking, alcohol consumption and smoking [21–26]. Medical condition such as induced abortion count, history of diabetes and hypertension, also lead to negative reproductive health outcomes [21, 27–30].

The aim of the present study is to describe how SES such as rural area, income, education and occupation, respectively, are associated with the risk of SA using baseline data from China Kadoorie Biobank, examining the effects of SES on SA through various methods, providing some implications for public health and further study.

## Methods

### Database

This study applied baseline data from China Kadoorie Biobank study, a large-scale population-based cross-sectional study which approached 0.5 million people from 10 geographically diverse areas of China. Details of the CKB study design and characteristics of the study participants have been described elsewhere [31, 32]. Briefly, 51, 2891 participants from the general population aged 30-79 years were included between 2004 and 2008 from 10 cities of China (5 urban areas consisting of Qingdao, Harbin, Liuzhou, Suzhou and Haikou and 5 rural areas consisting of Sichuan, Zhejiang, Hunan, Gansu, and Henan). Selection of the survey sites was based on local patterns of disease and exposure to certain risk factors, population stability, quality of death, and disease registries. Within each area, permanent residents without major disabilities in every 100–150 administrative units were sampled from local records, after identifying participants, we sent a letter to invite them to investigate. The questionnaire consisted of 10 major sections related to demographic and socioeconomic status, dietary and lifestyle habits, exposure to passive smoking and domestic indoor air pollution, medical history and current medication, physical activity, sleeping and mental status and reproductive history (for women). During the investigation, trained interviewers administered a standardized questionnaire by a laptop-based direct data-entry system, with built-in functions to prevent logical errors and missing items. The participation rate was 33% in rural areas and 27% in urban areas, and the main reasons for non-participant were absence from the home and reluctance to spend time visiting the screen center.

After a few weeks of baseline survey in each community, a quality control (QC) survey was done, involving ~3% of the participants randomly selected from the community with repeat questionnaire and measures on selected items. During the course of the survey, regular central monitoring was also undertaken to assess the recruitment rate, the distribution of certain key variables, and consistency of the data collected, both overall and by individual staff.

### Measurement

The present study mainly focused on spontaneous abortion as the outcome variable, which further dichotomized into “never happened”, and “happened once and above”. Exposure effect in this study primarily focused on one’s socioeconomic status which was indicated by residential area, household income, highest level of education and current occupation. Regarding to residential area, respondents from the following five provinces (Sichuan, Gansu, Henan, Zhejiang and Hunan) fell into the rural group, while those from Qingdao, Harbin, Haikou, Suzhou and Liuzhou fell into the urban group, for the strong association between rural/urban and SA. Annual household income was dichotomized into 2 groups: <20,000 yuan and ≥20,000 yuan. Highest level of education was categorized into 3 levels, primary school and below, middle school, high school and above. Current occupation was grouped into 6 types: agricultural and related workers, factory worker, professional worker (administrator/manager, professional/technical, sales and service workers included), housewife, unemployed and other or not stated (retired, self-employed, not stated included). Potential confounding variables were controlled in this study according to previous literature: tea consumption, alcohol consumption, cigarette smoking for lifestyle and induced abortion count, history of diabetes and hypertension for medical condition.

### Statistical analysis

As the information about SES in the baseline survey could not represent the SES during the time of pregnancy, in order to control the variance in baseline population, women aged <35 and >45 years old were excluded. Extreme values outside the 1 and 99% percentile of spontaneous abortion count were excluded during the univariate analysis. In addition, missing data were excluded from the analysis. When exclusion criteria applied, the total sample comprised of 84,531 women, 55% of them in rural and 45% in urban. In order to avoid the interference from the gestational diabetes mellitus and gestational hypertension, women with history of diabetes and hypertension were excluded.

Univariate analysis was applied to describe the means and standard deviation of continuous variables (age at study date, induced abortion count, and spontaneous abortion count) and percentages for categorical variables. In order to test for effect, modification with regards to rural/urban residence in the association between spontaneous abortion and socioeconomic status, women were stratified into two subgroups according to whether region was rural or not. Distribution of demographic and socioeconomic characteristics in SA was described.

Furthermore, logistic regression was used to examine the effect of SES on SA. Although the association between cigarette smoking and spontaneous abortion was non-significant, there was evidence of strong link between them in previous literature [33]. Odds ratios (OR) and 95% confidence interval (95% CI) were used to approximate associations between SES and spontaneous abortion. Multivariable logistic regression model was then used to assess the effect of important explanatory variables. Individual-level ORs were calculated for each category in comparison to a reference category, which was the first group in each category. For each model, observations with missing values for explanatory variables were excluded from the analysis. Crude ORs were calculated to describe the effect of SES on SA, individually. The multivariable model included all four indicators of SES (annual household income, highest level of education, current occupation and rural/urban areas), lifestyle (tea consumption, alcohol consumption and cigarette smoking) and induced abortion count. Likelihood ratio tests were used to compare the fit of model containing different variables, to test for departures from a linear trend in ordered categorical variables. Multivariable logistic regression analyses were performed within each subgroup, stratified by rural/urban residence, adjusting for all 4 confounders, namely tea consumption, alcohol consumption, cigarette smoking and induced abortion count. All statistical analyses were performed with the SAS software package V.9.4, setting *p*-value ≤0.05 as statistically significant.

### Ethical consideration

Prior to starting the project, central ethics approvals were obtained from Oxford University, and the China National CDC. In addition, institutional approvals were also obtained from institutional research boards at the local CDCs in the 10 regions.

## Results

In total, 302,632 women were recruited into the study at the first step. After excluding women aged <35 or >45 years old (215,845 women) and the extreme values of induced abortion count (459 women), 85,431 respondents were enrolled in the analyses (897 missing values were excluded). Table 1 shows the demographic, socioeconomic and reproductive characteristics of the sample from rural and urban areas, respectively.

**Table 1:** Demographic, socioeconomic and reproductive characteristics, by rural/urban

	Rural	Urban	Overall
Number of participants	47,150	37,381	84,531
Age, years (%)			
35–36	15.27	13.54	14.50
37–38	18.67	16.88	17.88
39–40	20.24	16.91	18.77
41–42	21.80	22.08	21.92
43–45	24.02	30.60	26.93
Mean (SD)	40.01 (0.94)	40.43 (3.03)	40.20 (2.99)
Annual household income, Yuan (%)			
<20,000	65.22	38.40	53.36
≥20,000	34.78	61.60	46.64
Highest level of education (%)			
Primary school and below	43.63	14.01	30.53
Middle school	44.28	36.62	40.89
High school and above	12.09	49.37	28.58
Current occupation (%)			
Agricultural and related workers	69.76	3.19	40.32
Factory worker	14.08	39.18	25.18
Professional worker	5.42	29.98	16.28
Housewife	6.60	6.31	6.47
Unemployed	0.41	10.86	5.03
Other or not stated	3.73	10.49	6.72
Spontaneous abortion count (%)			
0	90.96	96.25	93.30
1	7.29	3.02	5.40
≥2	1.75	0.73	1.30

### Socio-demographic characteristic

The mean (+SD) age was 40.20 (2.99) overall, 40.01 (0.94) in rural area and 40.43 (3.03) in urban area. The overall difference in annual household income between rural and urban was small. Whereas two-thirds respondents were from high-income households (annual household income ≥20,000 Yuan) in urban, only one-third were from high-income households in rural. Urban women tended to be better educated: one half of urban women completed high school, whereas only 12% of rural women did so. Regarding to occupation, most of rural women were agricultural worker and related workers (69.76%), but factory worker and professional worker took the majority (39.18 and 29.98%, respectively) in urban. About 7% women have experienced spontaneous abortion with the proportion being higher in rural (about 9%) compared with that of urban (about 4%).

### Factors associated with SA

Table 2 shows the crude adjusted odds ratio (COR) and adjusted OR (AOR) of SA, using multivariable regression model with all the potential confounders (when any category of socioeconomic factors was analyzed, the factor itself was excluded from the model) adjusted. Univariate analysis of comparison among all variables revealed that one’s residential area, i.e., urban or rural area, had the strongest association with SA; the likelihood of SA in rural was 2.56 times greater than in urban (COR = 2.56, 95%CI: 2.40–2.72). After adjusting all confounders, rural area still remained a strong association with SA (AOR = 1.68, 95%CI: 1.54–1.84). An inverse association was found between annual household income and the risk of SA. Women with high annual household income (>20,000 Yuan) had an AOR = 0.90 (95%CI: 0.84–0.97) of SA when compared with women with low annual household income (<20,000 Yuan). Same results also appeared in adjusted models. There was a remarkable ‘dose response’ of decreasing risk of SA along with the increasing levels of education in univariate analysis: compared with women who received education only from primary school and below, the COR of women with middle school education and above was 0.85 (95%CI: 0.80–0.90), and women with high school education and above was 0.53 (95%CI: 0.49–0.57). After adjusting other confounders, however, women with middle school education showed little difference with women with primary school education and below (AOR = 0.98, 95%CI: 0.92–1.04), whereas women with high school education and above still had a significantly protective effect in spontaneous abortion (AOR = 0.90, 95%CI: 0.82–0.98). As for effects of occupation on spontaneous abortion, the risk of SA was reduced in factory worker (COR = 0.37, 95%CI: 0.34–0.40), professional worker (COR = 0.39, 95%CI: 0.36–0.43), housewife (COR = 0.84, 95%CI: 0.76–0.94), unemployed women (COR = 0.41, 95%CI: 0.35–0.48) compared with agriculture and related workers; while after adjusting all confounders, the risk of spontaneous abortion was brought down significantly only in factory worker (AOR = 0.59, 95%CI: 0.53–0.66) and professional worker (AOR = 0.75, 95%CI: 0.66–0.84) compared with that of agriculture and related workers. Significant trends were found in income and education, heterogeneity were found in rural/urban and occupation (all *p* values < 0.05).

**Table 2:** The odds ratio of spontaneous abortion associated different socioeconomic status

	Women without SA *n* = 76,034	Women with SA *n* = 5,418	Crude OR (95% CI)	Adjusted OR (95% CI) ^a^
Region (%)				
Urban	45.64	24.73	1.00	1.00
Rural	54.36	75.27	2.56 (2.40–2.72)	1.68 (1.54–1.84)
*p* for difference ^b^			**<0.001**	**<0.001**
Annual household income, Yuan (%)				
< 20,000	52.68	64.89	1.00	1.00
≥ 20,000	47.32	35.11	0.60 (0.57–0.64)	0.90 (0.84–0.97)
*p* for trend^c﻿^			**<0.001**	**0.003**
Highest level of education (%)				
Primary school and below	30.05	37.49	1.00	1.00
Middle school	40.72	43.13	0.85 (0.80–0.90)	0.98 (0.92–1.04)
High school and above	29.23	19.38	0.53 (0.49–0.57)	0.90 (0.82–0.98)
*p* for trend ^c^			**<0.001**	**0.033**
Current occupation (%)				
Agricultural worker and related workers	39.17	59.06	1.00	1.00
Factory worker	25.95	14.29	0.37 (0.34–0.40)	0.59 (0.53–0.66)
Professional worker	16.79	9.93	0.39 (0.36–0.43)	0.75 (0.66–0.84)
Housewife	6.26	7.95	0.84 (0.76–0.94)	1.11 (0.99–1.24)
Unemployed	5.11	3.14	0.41 (0.35–0.48)	0.84 (0.71–1.01)
Other or not stated	6.72	5.63	0.56 (0.49–0.63)	0.95 (0.83–1.09)
*p* for heterogeneity ^d^			**<0.001**	**0.008**

*NB* The COR and AOR in the text represent the crude OR and adjusted OR in the table, respectively

^a^ Adjustments: rural/urban, income, education, occupation, tea consumption, alcohol consumption, smoking status, induced abortion count

^b^ *p* for difference was used to test the difference between rural and urban area as binary variable

^c^ *p* for trend was used to test the significance of trend on ordinal categorical variables like income and educational level

^d^ *p* for heterogeneity was used to test the heterogeneity between occupation as unordered categorical variables

Bold values signify significant findings *p* <0.05

### Stratification analysis by residence

**Fig. 1:**
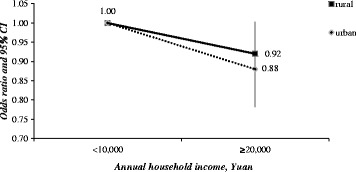
Odds ratio for spontaneous abortion by annual household income, by rural/urban

Because of the strong association between the type of residence (urban/rural) and SA (AOR = 1.68, 95%CI: 1.54–1.84, *p* < 0.001), a stratified analysis was applied here by rural and urban area, respectively. In both subgroups, the risk of SA was lower in women with high annual household income (rural: A OR = 0.92, 95%CI: 0.84–1.00; urban: AOR = 0.88, 95%CI: 0.78–0.99) compared with women with low annual household income. Comparison of AORs for SA within two subgroups by annual household income was presented in Fig. 1.

**Fig. 2:**
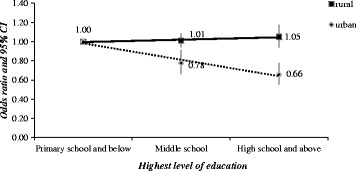
Odds ratio for spontaneous abortion by highest level of education, by rural/urban

Risk effect for education on spontaneous abortion was found for women in urban but not for women in rural (rural: AOR = 0.78, 95%CI: 0.66–0.91 for middle school, AOR = 0.66, 95%CI: 0.55–0.78 for high school and above; urban: AOR = 1.01, 95%CI: 0.94–1.09 for middle school, AOR = 1.05, 95%CI: 0.34–1.17 for high school and above). Fig. 2 presents the comparison between AORs of SA within two subgroups by highest level of education.

As for the effect of occupation on SA, there was no difference in both two subgroups. Significant rends were found in income and education, and significant heterogeneities in rural/urban and occupation in urban (see Additional file 1). However, there was no clear trend in income or education and no clear heterogeneity in rural/urban or occupation for women in the rural (see Additional file 2).

## Discussion

This study utilized the baseline data from the China Kadoorie Biobank study (CKB), exploring the possible association between SES and SA. It was found in this study that the risk of spontaneous abortion in rural was higher than of urban. Women with high income had a lower risk of SA when compared with women with low income. Compared with women with primary school education and below, the risk of SA in women with middle school education or above was lower. The risk of SA only reduced in factory worker and professional worker compared with agriculture and related workers. After stratifying by rural/urban, the association between income and SA in urban was stronger than that of rural. Association between education and SA was found in urban but not in rural and there was no difference between the two subgroups with respect to the occupation variable.

### Occurrence of spontaneous abortion

12–15% of clinically recognized pregnancies were reported culminating as SA [34]. In China, the prevalence of SA was 6%–14% [35, 36], whereas the prevalence in this study was 6.70% which was close to the lower boundary. It was consistent with previous studies although slightly different from clinical data, which could be explained by limited access to a true data of SA cases, as well as latent cases not taken into account. Most cases of SA occur in early stages of pregnancy and a certain proportion does not result to medical intervention or hospitalization.

### Spontaneous abortion and socioeconomic status

The present study found that compared with women with annual household income less than 20,000 Yuan, women with annual household income more than 20,000 Yuan were less likely to experience SA (AOR = 0.90, 95%CI: 0.84–0.97). It was similar with the point from a study from U.S, who reported that a history of spontaneous abortion was significantly related to income below poverty (AOR = 1.34, 95%CI: 1.06–1.70) [14]. Psychological evidence pointed out that decreased wealth could lead to increased mental stress [37]. Low income and material deprivation in general was associated with poor housing, nutrition, and healthcare access, which in turn negatively affected the general health physically and mentally [38, 39]. Nevertheless, there are differences that exist in a bigger context: most of the population in developed countries was relatively homogeneous in terms of income levels as compared with income levels of other populations [40]. Therefore, an association would be more evident in countries like China where a greater distinction in living circumstances existed between rich and poor [41]. However, the classification of income groups in this study was binary which may lose some information regarding to different income levels.

The present study also found that compared with women with low educational attainment, women with high educational attainment enjoyed a decreased risk of SA (AOR = 0.90, 95%CI: 0.82–0.98) exemplifying the marked dose-response relationship. Results from previous literatures have confirmed that education was associated with the incidence of SA [20]. An early study calculated adjusted odds ratios (AORs) for spontaneous abortion were 0.9 (95%CI: 0.6–1.4) and 0.6 (95%CI: 0.4–0.9) for women reporting 7–11 and ≥ 12 years of schooling, respectively, compared with women reporting < 7 years of education [42]. Educational level has been well established as a good indicator of social position. Higher level of education is associated with better health through nurturing a healthier life style [43]. Preventive uptake and successful use of preventive and curative care, as well as stress confrontation are also dependent on the level of educational attainment [44, 45].

In this study, the risk of SA reduced in factory workers (AOR = 0.59, 95%CI: 0.53–0.66) and professional worker (AOR = 0.75, 95%CI: 0.66–0.84) compared with the risk of SA in agriculture and related workers. Several domestic studies, however, showed similar results with present study [4, 13]. In contrast to such findings, previous studies have concluded that occupation was not associated with spontaneous abortion. Alan E et al. from U.S. reported that neither individual nor combine work-related or home-related physical exertion measures were associated with an increased risk of SA [46]. However, a number of differences between these two studies, including study design and the population studied, might have contributed to the lack of consistency between the results. Such study collected prospective detailed data on physical exertion as well as data on potential confounders of a large group including women employed in a variety of occupations, whereas the present study only questioned respondents’ current occupation classifying it into 5 categories. Several studies [15, 18, 47] also reported that occupation was not associated with SA, with possible explanation being that in developed countries there was a high social security for people in labor market; another speculation could be that different occupation posed same risks and these risks outweighed each other.

### Differences between rural and urban

Health outcomes vary among areas due to numerous factors, such as: social background (e.g., quality living environment, education, access to community networks, and social welfare), economic development (e.g., levels of employment, Gross Domestic Product per capita, and working and environmental conditions), individual behaviors (e.g., lifestyle, nutrition), as well as access to healthcare [43, 45]. Due to implementations of *Program of China’s Women Development (in Chinese)* and *Program of China’s Children Development (in Chinese)*, the gap between maternal mortality rates (per 1,000 thousands population) decreased gradually in the last decade, from 46.3 in urban vs. 100 in rural, to 22.2 vs. 25.6 [48, 49]. Nevertheless, an evident unbalanced distribution of health workforce between rural and urban areas still exists in China. According to the *China Health Statistical Yearbook 2013 (in Chinese)*, health workforce in Chinese rural area was 3.41 health workers per 1,000 population, while that in the urban area was as many as 8.54 health workers per 1000 population [50]. Women in rural areas still face more reproductive health challenges compared to their urban fellows. Based on this speculation, a stratification analysis of rural and urban was taken. After stratifying by rural and urban, differences were revealed between rural areas and urban areas. The prevalence of spontaneous abortion was 9.04% in rural and 3.75% in urban. Both in rural and urban areas, risk of SA were lower in women with high annual household income, compared with those with low annual household income. As Fig. 1 presented, association of annual household income on urban women was stronger than that of rural women in this study. Otherwise, in the present study, effect of educational attainment on SA was found only in women from urban. One possible explanation for these results was that excluding the variables studied in present study, perinatal health was closely related to health workforce and medical resource in local areas. Compared with those from the urban area where different socioeconomic status could access similar healthcare and thus better health equity, women in rural areas suffered mainly from a limited access to appropriate healthcare, regardless of their socio-economic status. Previous literature also revealed that living in an urban neighborhood had favorable perinatal health related effects than rural, for that living in rural neighborhood elicits problems such as smoking, drugs or alcohol use, physical inactivity, and unhealthy food patterns [29].

### Strengths and limitations

This study has several strengths significant to note. Firstly, the database utilized the baseline data from CKB, a large prospective study recruiting and assessing 0.5 million people. It was able to provide estimates with substantial power because such a large study sample provided a high number of spontaneous abortion. Secondly, the association between SES and SA was stratified by rural/urban to explore differences between rural areas and urban areas, which was a novel strategy rarely used in previous studies. This study also has some potential limitations: firstly, this study was a cross-sectional study, so it was not possible to make causal inference between the SES and SA. More prospective evidence should be provided in order to do so. Secondly, the SES in this cross-sectional study could not represent the SES during the time of pregnancy. Most importantly, women aged <35 and >45 years old were excluded altogether.

## Conclusions

This study is an analysis of baseline data from the China Kadoorie Biobank study, revealing that spontaneous abortion remains the most common adverse outcomes of pregnancy. Researchers found significant associations between women’s SES and the risks of SA: Lower income and educational attainment were inversely associated with the risk of SA. Women with agricultural and related work had a significantly higher prevalence of SA, which indicated that general women with low SES had higher risk of SA. Furthermore, the associations between SES and SA differed between rural and urban areas: the association between income and SA in urban was stronger than rural, association between education and SA was found in urban but not in rural and there was no difference in occupation between two subgroups. It provides important implications for policy and program development that women with low SES need more attention, support and observation on health promotion strategies. In addition, further studies are needed to understand the underlying psychosocial mechanisms of the association.

## Additional files

Additional file 1: Table S1. The odds ratio of spontaneous abortion associated different socioeconomic status, by urban. (DOCX 14 kb)
Additional file 2: Table S2. The odds ratio of spontaneous abortion associated different socioeconomic status, by rural. (DOCX 14 kb)

## Abbreviations

95%CI: 95% confidence interval
AOR: Adjusted odds ratio
COR: Crude odds ratio
OR: Odds ratio
QC: Quality control
SA: Spontaneous abortion
SD: Standard deviation
SES: Socioeconomic status
