# Supplementary material for: Factors associated with spontaneous abortion: a cross-sectional study of Chinese populations
Source: Reprod Health. 2017 Mar 4;14:33. doi: 10.1186/s12978-017-0297-2 (PMC5336639; doi:10.1186/s12978-017-0297-2)
Supplement: Additional file 1: Table S1. — The odds ratio of spontaneous abortion associated different socioeconomic status, by urban. (DOCX 14 kb) [file 12978_2017_297_MOESM1_ESM.docx]

**Additional file 1: Table S1. The odds ratio of spontaneous abortion associated different socioeconomic status, by urban**

|  | No. of cases | Prevalence (%) | Crude OR  (95% CI) | Adjusted OR  (95% CI)^‡^ | |
| --- | --- | --- | --- | --- | --- |
| **Annual household income, Yuan** | | | | | |
| <20,000 | 590 | 1.64 | 1.00 | 1.00 | |
| ≥20,000 | 750 | 2.08 | 0.78 (0.70-0.87) | 0.88 (0.78-0.99) | |
| ***p* for trend** | | | ***p*<0.001** | ***p*=0.036** | |
| **Highest level of education** | | | | | |
| Primary school and below | 269 | 0.75 | 1.00 | 1.00 | |
| Middle school | 504 | 1.40 | 0.71 (0.61-0.83) | 0.78 (0.66-0.91) | |
| High school and above | 567 | 1.57 | 0.59 (0.51-0.68) | 0.66 (0.55-0.78) | |
| ***p* for trend** | | | ***p*<0.001** | ***p*<0.001** | |
| **Current occupation** | | | | |  |
| Agricultural worker & related workers | 83 | 0.23 | 1.00 | 1.00 | |
| Factory worker | 432 | 1.20 | 0.42 (0.33-0.53) | 0.56 (0.43-0.74) | |
| Professional worker | 372 | 1.03 | 0.47 (0.37-0.60) | 0.71 (0.54-0.94) | |
| Housewife | 115 | 0.32 | 0.71 (0.53-0.95) | 0.94 (0.70-1.28) | |
| Unemployed | 161 | 0.45 | 0.57 (0.44-0.75) | 0.83 (0.62-1.10) | |
| Other or not stated | 177 | 0.49 | 0.66 (0.50-0.86) | 0.91 (0.69-1.21) | |
| ***p* for heterogeneity** |  |  | ***p*<0.001** | ***p*<0.001** | |

‡: Adjustments: income, education, occupation, tea consumption, alcohol consumption, smoking status, induced abortion count
