# Supplementary material for: Factors associated with spontaneous abortion: a cross-sectional study of Chinese populations
Source: Reprod Health. 2017 Mar 4;14:33. doi: 10.1186/s12978-017-0297-2 (PMC5336639; doi:10.1186/s12978-017-0297-2)
Supplement: Additional file 2: Table S2. — The odds ratio of spontaneous abortion associated different socioeconomic status, by rural. (DOCX 14 kb) [file 12978_2017_297_MOESM2_ESM.docx]

**Additional file 2: Table S2. The odds ratio of spontaneous abortion associated different socioeconomic status, by rural**

|  | No. of cases | Prevalence (%) | Crude OR  (95% CI) | Adjusted OR  (95% CI)^‡^ | |
| --- | --- | --- | --- | --- | --- |
| **Annual household income, Yuan** | | | | | |
| <20,000 | 2,926 | 6.44 | 1.00 | 1.00 | |
| ≥20,000 | 1,152 | 2.54 | 0.73 (0.68-0.78) | 0.92 (0.84-1.00) | |
| ***p* for trend** | | | ***p*<0.001** | ***p*=0.057** | |
| **Highest level of education** | | | | | |
| Primary school and below | 1,762 | 3.88 | 1.00 | 1.00 | |
| Middle school | 1,833 | 4.04 | 1.02 (0.96-1.10) | 1.01 (0.94-1.09) | |
| High school and above | 483 | 1.06 | 1.00 (0.90-1.11) | 1.05 (0.94-1.17) | |
| ***p* for trend** | | | ***p*<0.001** | ***p*=0.446** | |
| **Current occupation** | | | | |  |
| Agricultural worker & related workers | 3,117 | 6.86 | 1.00 | 1.00 | |
| Factory worker | 342 | 0.75 | 0.52 (0.47-0.59) | 0.61 (0.54-0.70) | |
| Professional worker | 166 | 0.37 | 0.67 (0.57-0.79) | 0.78 (0.66-0.93) | |
| Housewife | 316 | 0.70 | 1.11 (0.98-1.26) | 1.12 (0.99-1.28) | |
| Unemployed | 9 | 0.02 | 0.47 (0.24-0.92) | 0.57 (0.29-1.11) | |
| Other or not stated | 128 | 0.28 | 0.76 (0.63-0.91) | 0.87 (0.72-1.06) | |
| ***p* for heterogeneity** |  |  | ***p*<0.001** | ***p*=0.503** | |

‡: Adjustments: income, education, occupation, tea consumption, alcohol consumption, smoking status, induced abortion count
